# Interleukin-7 and Toll-Like Receptor 7 Induce Synergistic B Cell and T Cell Activation

**DOI:** 10.1371/journal.pone.0094756

**Published:** 2014-04-16

**Authors:** Angela Bikker, Aike A. Kruize, Kim M. G. van der Wurff-Jacobs, Rogier P. Peters, Marije Kleinjan, Frank Redegeld, Wilco de Jager, Floris P. J. G. Lafeber, Joël A. G. van Roon

**Affiliations:** 1 Department of Rheumatology & Clinical Immunology, University Medical Centre Utrecht, Utrecht, the Netherlands; 2 Department of Pharmaceutical Sciences, Faculty of Science, Utrecht University, Utrecht, the Netherlands; 3 Department of Pediatric Immunology, University Medical Centre Utrecht, Utrecht, the Netherlands; Emory University School of Medicine, United States of America

## Abstract

**Objectives:**

To investigate the potential synergy of IL-7-driven T cell-dependent and TLR7-mediated B cell activation and to assess the additive effects of monocyte/macrophages in this respect.

**Methods:**

Isolated CD19 B cells and CD4 T cells from healthy donors were co-cultured with TLR7 agonist (TLR7A, Gardiquimod), IL-7, or their combination with or without CD14 monocytes/macrophages (T/B/mono; 1 : 1 : 0,1). Proliferation was measured using ^3^H-thymidine incorporation and Ki67 expression. Activation marker (CD19, HLA-DR, CD25) expression was measured by FACS analysis. Immunoglobulins were measured by ELISA and release of cytokines was measured by Luminex assay.

**Results:**

TLR7-induced B cell activation was not associated with T cell activation. IL-7-induced T cell activation alone and together with TLR7A synergistically increased numbers of both proliferating (Ki67^+^) B cells and T cells, which was further increased in the presence of monocytes/macrophages. This was associated by up regulation of activation markers on B cells and T cells. Additive or synergistic induction of production of immunoglobulins by TLR7 and IL-7 was associated by synergistic induction of T cell cytokines (IFNγ, IL-17A, IL-22), which was only evident in the presence of monocytes/macrophages.

**Conclusions:**

IL-7-induced CD4 T cell activation and TLR7-induced B cell activation synergistically induce T helper cell cytokine and B cell immunoglobulin production, which is critically dependent on monocytes/macrophages. Our results indicate that previously described increased expression of IL-7 and TLR7 together with increased numbers of macrophages at sites of inflammation in autoimmune diseases like RA and pSS significantly contributes to enhanced lymphocyte activation.

## Introduction

Interleukin-7 (IL-7) is a potent T cell activating cytokine that causes proliferation, survival and differentiation of T cells in the periphery to maintain homeostatic T cell balance [1]. Not only in health, but also in disease, IL-7 has been shown to play an important role in T cell expansion and enhancement of T cell-driven immunity. Addition of IL-7 increases T cell numbers and functionality in immunodeficient states due to HIV infection, chemotherapy, and after bone marrow transplantation [2], [3], [4].

Furthermore, IL-7 and its receptor have been implicated in several autoimmune diseases like rheumatoid arthritis (RA) [5], [6], [7], psoriasis [8], spondylarthritis [9], inflammatory bowel’s disease (IBD)[10], [11] multiple sclerosis (MS) [12], [13], [14], and recently primary Sjögren’s Syndrome (pSS) [15], [16]. In the inflamed tissues of patients with autoimmune diseases, increased IL-7 production and IL-7 receptor (IL-7R) expression by tissue cells and immune cells have been documented [5], [6], [7], [8], [9], [15], [16]. In many *in vitro* models IL-7 was shown to induce T cell activation (Th1 and Th17 induction) and T cell-dependent activation of monocytes/macrophages and dendritic cells (DCs) [5], [15], [17]. In addition, gene polymorphisms of the IL-7Rα gene are associated with susceptibility to MS [13]. Finally, IL-7 and IL-7R have been shown to play critical proinflammatory roles in experimental models for diabetes, MS, IBD and RA [3],[14],[18],.

Although its role on T cell activation has extensively been studied (reviewed in [21], [22]), less is known about the stimulatory effect of IL-7 on B cells. Although reduced serum immunoglobulin levels in IL-7R-deficient individuals suggested that IL-7 might play a role in activation of mature human B cells [23], direct evidence for this is lacking. Recently, we found that, at least *in vitro*, IL-7 significantly and equally effective increased B cell activation in PBMC culture and CD4 T cell co-culture of healthy controls and patients with pSS [24]. Another recently published study demonstrated that IL-7 modulates survival and activation of B cells from healthy controls trough CD70 expression by T cells [25]. In the present study it was investigated to what extent IL-7-driven T cell-dependent B cell activation could synergize with TLR7-driven B cell activation.

Pathogen-associated molecular pattern (PAMP) -recognizing molecules such as Toll-like receptors (TLRs) have extensively been examined and have been implicated to play an important role in autoimmune diseases like RA [26], [27], systemic sclerosis [28], systemic lupus erythematosus (SLE) [29], and pSS [30], [31], [32], [33]. Because of the predominance of autoantibodies directed against RNA-binding proteins (anti-Ro and La), in several systemic autoimmune diseases TLR7 has been studied. TLR7 is located intracellular, in the endosomal compartments of B cells and plasmacytoid DCs (pDCs) [34], [35]. TLR7 is specialized in recognizing single-stranded RNA (ssRNA) originating from viruses [34], but it has been postulated that self-RNA becomes a target, when self-tolerance is breached [36]. This break of tolerance has been demonstrated upon EBV infection, inducing anti-Ro and Sm antibodies [37], [38]. In autoimmune diseases, autoantibodies directed against RNA-binding self-antigens (anti-Ro/SSA and La/SSB), consequently deliver aberrantly exposed extracellular RNA to the endosomal compartment of B cells and pDCs (via the B cell receptor or Fc receptors) and subsequently trigger TLR7 signalling [39].

TLR7 and IL-7 (eg. upon viral triggering) could be involved in the early stages of B cell immunopathology of several systemic autoimmune diseases, this study aimed to investigate the hypothesis of synergistic B and T cell activation upon TLR7-driven B cell and IL-7-driven T cell stimulation. In addition, increased numbers of monocytes/macrophages are present at sites of inflammation and activated monocytes (eg. with enhanced IFN signature) are present in the circulation of systemic autoimmune diseases [40], [41]. Given the importance of myeloid cells in immunopathology of many rheumatic diseases like RA, the capacity of monocytes/macrophages to potentiate this effect was examined to reveal their potential contribution to TLR7/IL-7-driven immune activation in systemic autoimmune diseases. In this respect the ratio of T and B cells and monocytes/macrophages (1∶1∶0,1) used in this study was chosen similar to that observed at sites of inflammation, such as in the labial salivary gland of pSS patients.

## Methods

### Ethics Statement

Peripheral blood from healthy donors was used in this study. They have given a written consent to participate in the in-house donor program, approved by the UMCU medical ethical committee. This study has been approved by the UMCU medical ethical committee.

### Cell isolation and culture

Peripheral blood (PB) from healthy donors was diluted 1∶1 with RPMI 1640 medium (Gibco BRL, Life Technologies, Belgium) containing penicillin (100 U/ml), streptomycin (100 µg/ml) and glutamine (2 mM) (PSG). Peripheral blood mononuclear cells (PBMCs) were isolated by density centrifugation using Ficoll-Paque (Pharmacia, Uppsala, Sweden). CD19 B cells, CD14 monocytes/macrophages and CD4 T cells were isolated from PBMCs by magnetic-activated cell sorting (MACS). CD19 cells and CD14 monocytes/macrophages were positively selected by incubating PBMCs with anti-CD19 or anti-CD14 labelled with goat anti-mouse IgG magnetic beads (Miltenyi Biotec, Utrecht, The Netherlands) following MACS procedures according to manufactures instructions using the autoMACS (Miltenyi Biotec). Untouched CD4 T cells were negatively selected by using the CD4 isolation kit II (Miltenyi Biotec) and autoMACS (Miltenyi Biotec).

CD4 T cells and CD19 B cells were co-cultured 1∶1 in 96-wells plates and proliferation determined by ^3^H-thymidine incorporation during the last 18 hours of culture (5 µCi/ml, NEN Life Science Products, Amsterdam, The Netherlands). Additionally, proliferation and activation of B and T cells was determined by surface activation marker expression and intracellular Ki67 expression, respectively (24-wells plate). Cells were cultured for 4 days at a concentration of 5.10^5^ cells/ml at 37°C. For analysis of cytokine and immunoglobulin levels, after a 6− and 12−days culture period respectively, supernatants were rendered cell free and stored at -80°C until analysis. In addition, all analyses were also performed for CD4 T/CD19 B cell co-cultures in the presence of CD14 monocytes/macrophages (5.10^4^ cells/ml, 1∶1∶0,1 ratio). Cells were cultured in RPMI/glutamax (Gibco BRL) with additional penicillin (100 U/ml, Yamanouchi), streptomycin (100 µg/ml Fisiopharma), and 10% fetal calf serum (FCS, Gibco BRL) in the presence or absence of 1 ng/ml TLR7 agonist (Gardiquimod, Invivogen, San Diego, CA, USA) and 10 ng/ml IL-7 (Peprotech Inc., Rocky Hill, NY, USA). An increase in proliferation and activation/maturation is regarded as additive when the combination of stimulations is the sum, and synergistic when the combination of stimulations is higher than the sum of the individual stimulations (minus the value of the unstimulated control condition).

### Markers of activation, proliferation, and B cell differentiation by flow cytometry

Expression of the following markers on CD4 T cells and CD19 B cells was assessed after 4 days in culture using the following monoclonal antibodies (mAb): CD4-PerCP (Biolegend, San Diego, CA, USA), CD19-APC (BD, Erembodegem-Aalst, Belgium), CD25-PE (DAKO, Glostrup, Denmark), and HLA-DR-FITC (BD). For analysis of B cell differentiation the following monoclonal antibodies were used: CD3-FITC (BD Pharmingen, San Diego, CA, USA), CD19-APC-Alexa Fluor750 (BD), CD20-eFluor450 (eBioscience, San Diego, CA, USA) CD27-APC (ImmunoTools GmbH, Friesoythe, Germany), CD38-PE-CY7 (BD), and IgD-PE (BD). IgG1-FITC/IgG1-PE (Immunotech, Marseille, France) was used as isotype control.

Ki67 intracellular staining was performed using mouse-anti-human Ki67-FITC staining set (eBioscience) following manufactures instructions. Cell acquisition was done using a FACScan flow cytometer and data were analysed with FlowJo software, version 7.5 (Tree Star Inc., Oregon, USA).

### Immunoglobulin and cytokine measurements

IgG, IgM were measured by ELISA according to the manufacturer's instructions (Bethyl laboratories, Montgomery, TX, USA), kappa and lambda free light chains (FLCs) were measured with in-house developed ELISA kits adapted from Abe et al. [42], and described elsewhere [43]. IL-6 levels were determined by ELISA according to the manufacturer’s instructions (Biosource, Etten-Leur, The Netherlands; detection limit 10 pg/ml). All other cytokines (IFNγ, IL-2, IL-4, IL-10, IL-17A, IL-21, IL-22) were determined by a multiplex cytokine assay as extensively described elsewhere [44].

### Statistical analysis

Culture conditions were analysed by the parametric paired sample *t* test or the nonparametric Wilcoxon’s singed rank test where appropriate. All statistical analyses were performed using Graphpad Prism (GraphPad Prism 5.0, GraphPad software Inc.) and differences with a p-value of 0.05 or less were considered statistically significant.

## Results

### TLR7 and IL-7 synergistically increase proliferation of B cells in co-culture with CD4 T cells

**Figure 1:**
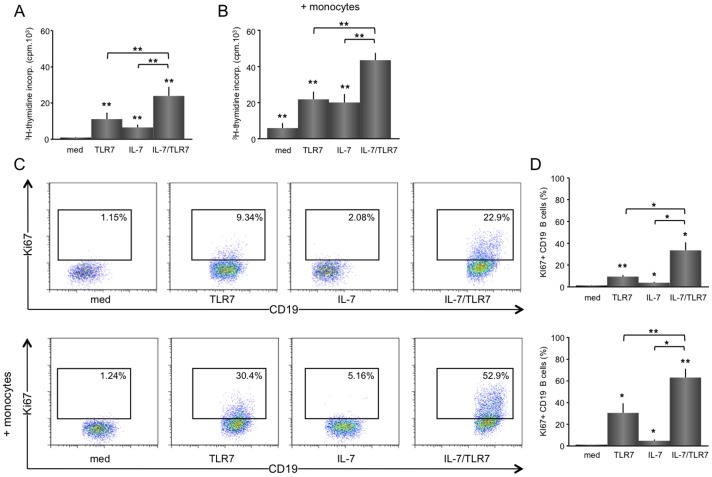
IL-7 synergistically increases proliferation of TLR7-stimulated B cells in co-culture with CD4 T cells, which is enhanced by monocytes/macrophages. Isolated B cells co-cultured 1∶1 (5.10^5^ each) together with CD4 T cells for 6 days show an increased lymphocytic proliferation upon TLR7 or IL-7 stimulation, which is additively increased upon combined stimulation with IL-7/TLR7 (n = 8) (A). A similar, but overall enhanced effect for the total proliferation is seen when monocytes/macrophages are added to the culture (5.10^4^ B). Representative FACS stainings for KI67^+^ B cells from an unstimulated, TLR7, IL-7, and TLR7/IL-7 stimulated CD4 T/B cell co-culture -/+ monocytes/macrophages are shown as well as the average data (n = 5) (C, D). TLR7 induces a significant increase in the percentage of KI67^+^ B cells. IL-7 stimulation induces a small, but statistically significant increase in Ki67^+^ B cells. When TLR7 and IL-7 are added together a synergistic increase in proliferation is observed (C). Overall the effects are enhanced by addition of monocytes/macrophages to the T/B cell co-cultures (D). * and ** indicate a statistical significant differences of p<0.05 and p<0.01, respectively, as compared to medium values or between treatments.

In line with the absence of TLR7 in T cells and the IL-7R on B cells, T cells only responded to IL-7 and B cells only to TLR7 stimulation, albeit at a much lower level (data not shown). IL-7R expression was measured on all populations before and after stimulation. The receptor was only expressed on T cells and rapidly down regulated upon activation by IL-7. IL-7R was not expressed on B cells and monocytes and surface expression was not detected on these cells after stimulation. (data not shown). Lymphocyte proliferation of T cell/B cell co-cultures as measured by ^3^H-thymidine incorporation was significantly increased by TLR7 (mean ±SEM; from 818 ±256 cpm to 10970 ±3683 cpm, p<0.01), IL-7 (to 6430 ±1597 cpm, p<0.01) and additively by TLR7 plus IL-7 (to 23901 ±5080 cpm, p<0.01 *vs.* cultures with IL-7 or TLR7 alone) (fig. 1A). Monocytes/macrophages added to the T/B cell co-cultures significantly enhanced TLR7 (from 5884 ±2776 cpm to 20081 ±4724 cpm, p<0.01), IL-7 (to 21853 ±4241 cpm, p<0.001) and IL-7/TLR7-induced (to 43613 ±4090 cpm, p<0.01) proliferation, but no significant change in the proliferation pattern was observed (fig. 1B).

TLR7 specifically induced B cell activation, as measured by KI67^+^ (from 1.2 ±0.2% to 9.3 ±1.4%, p<0.01). Whereas IL-7 on its own only slightly increased B cell proliferation (from 1.2 ±0.2% to 3.7 ±0.7%, p<0.05), the combination of IL-7 and TLR7 synergistically increased the number of Ki67^+^ B cells (33.4 ±7.3%) (fig. 1C). Furthermore, TLR7-induced B cell proliferation (30.2 ±8.9%, p = 0.07) and TLR7/IL-7-induced stimulation (63.0 ±8.0%, p<0.01) (fig. 1D) was enhanced by monocytes/macrophages. The average cell viability for these cell cultures was 75.4 ±2.4% for the unstimulated, 76.9 ±3.1% for TLR7-stimulated, 77.3 ±1.3% for IL-7-stimulated and 77.0 ±2.9% for IL-7/TLR7-stimulated cultures in the absence of monocytes/macrophages (n = 8). In the presence of monocytes/macrophages the average cell viability was 82.3 ±1.8% for the unstimulated, 76.4 ±2.5% for TLR7-stimulated, 79.7 ±2.1% for IL-7-stimulated and 76.6 ±2.7% for IL-7/TLR7-stimulated cultures (n = 8). No statistically significant differences were found between the different stimuli and with or without monocytes/macrophages.

### TLR7 and IL-7 synergistically increase CD4 T cell proliferation in co-cultures of T cells and B cells

**Figure 2:**
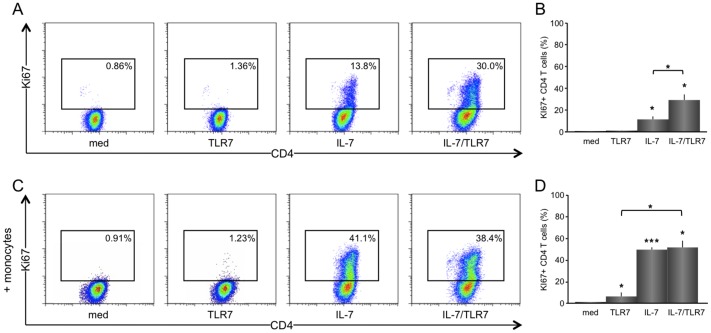
IL-7 and TLR7 synergistically increases CD4 T cell proliferation in T and B co-cultures. Representative FACS stainings for KI67^+^ CD4 T cells from unstimulated, IL-7, and TLR7/IL-7 stimulated T/B cell co-cultures in the absence or presence of monocytes/macrophages are shown (n = 5). IL-7 significantly stimulates proliferation of CD4 T cells, which is synergistically increased when combined with TLR7 stimulation (**A, B**). IL-7-induced CD4 T cell proliferation is enhanced in the presence of monocytes/macrophages, but no additive effect is observed with a combination of IL-7 and TLR7 stimulation (**C, D**).*p<0.05 and **p<0.001 indicate statistical significance compared to medium values.

TLR7-induced B cell activation in CD4 T/B cell co-cultures was not associated with T cell activation (mean ±SEM; from 0.8 ±0.1% to 1.1 ±0.3%), whereas IL-7 significantly increased CD4 T cell proliferation (from 0.8 ±0.1% to 11.5 ±2.8%, p<0.05), and IL-7 added to TLR7 also synergistically increased T cell proliferation (29.2 ±5.2%, p<0.05) (fig. 2A, representative; fig. 2B, average). Monocytes/macrophages strongly enhanced proliferation of CD4 T cells upon IL-7 stimulation (from 11.5 ±2.8% to 49.3 ±2.2%, p<0.001), equally effective to the combination of IL-7 and TLR7 stimulation (51.4 ±6.3%) (fig. 2C, representative; fig. 2D, average).

### IL-7 additively up-regulate expression of CD19 and HLA-DR on B cells

**Figure 3:**
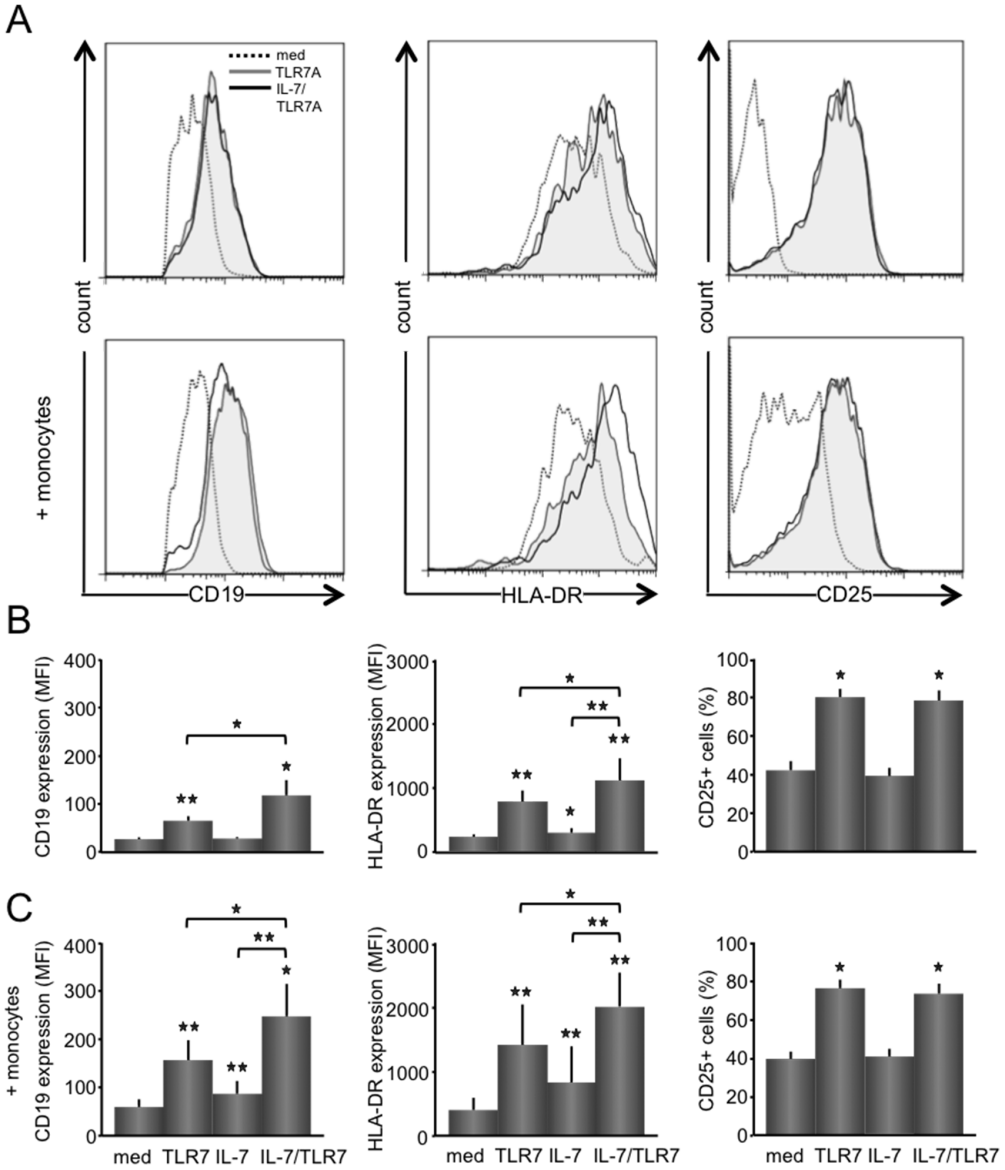
IL-7 enhances the up-regulated expression by TLR7 of CD19 and HLA-DR on B cells. Representative histograms for CD19, HLA-DR, and CD25 expression on B cells from unstimulated, TLR7, and IL-7/TLR7 stimulated T/B cell co-cultures are shown (n = 8) (**A**). TLR7 causes a significant increase in CD19 and HLA-DR B cell expression and in the number of CD25^+^ B cells. IL-7 stimulation induces a slight, but significant increase in HLA-DR expression, but with the addition of IL-7 to TLR7 stimulation a significant and increased expression is seen for CD19 and HLA-DR on B cells (**B**). Monocytes/macrophages added to the T and B cell co-cultures, significantly enhanced overall expression levels, displaying a similar relative expression pattern as seen without monocytes/macrophages (**C**). *p<0.05, **p<0.01 indicate a statistical significant difference compared to medium values.

Associated with increased B cell proliferation upon IL-7 and TLR7 stimulation, similar increases were observed for the expression of maturation and activation markers on B cells (CD19, HLA-DR, and CD25) and on T cells (HLA-DR, and CD25) (fig. 3A, representative histograms). TLR7 stimulation up regulated expression of CD19 (mean ±SEM; MFI from 26.8 ±3.3 to 63.4 ±9.6, p<0.01) and HLA-DR (MFI from 240 ±39 to 791 ±169, p<0.01) on B cells and increased percentages of CD25^+^ B cells (from 42.2 ±4.8% to 80.1 ±4.4%, p<0.05). The combined stimulation with IL-7 and TLR7 enhances up-regulation of CD19 expression (MFI 116 ±30, p<0.05 as compared with TLR7 and IL-7 stimulations alone) and HLA-DR expression (MFI 1121 ±341, p<0.05 as compared with TLR7 and IL-7 stimulations alone). No difference in increase between TRL7 and IL-7/TLR7 was seen for the percentages of CD25^+^ B cells. When monocytes/macrophages were added to CD4 T/CD19 B cell co-cultures similar but in general higher expression patterns of CD19 and HLA-DR upon stimulation were observed (fig. 3B and C).

Furthermore, to assess the effect on B cell differentiation in two donors, differentiation markers CD19, CD20, CD27, IgD, CD38 were analysed by flow cytometry. Within the total B cell population, an additive effect of IL-7 and TLR7 stimulation on the increase of CD27^+^IgD^−^ memory B cells (medium, 3.3 ±0.7% *vs*. IL-7, 3.4 ±0.7% *vs*. TLR7, 10.6 ±3.0% *vs.* IL-7/TLR7, 14.0 ±5.3%) and a slightly enhanced increase of CD27^high^CD38^high^ (CD19^+^CD20^−^) plasmablasts (medium 0% *vs*. IL-7 0% *vs*. TLR7 2.6 ±2.3% *vs.* IL-7/TLR7 4.1 ±3.3%) was seen (data not shown). This effect was most prominent in the presence of monocytes/macrophages (medium, 9.8 ±2.9% *vs*. IL-7, 6.6 ±1.8% *vs*. TLR7, 17.8 ±7.0% *vs.* IL-7/TLR7, 28.1 ±12.5% (memory B cells) and 5.7 ±5.1% *vs.* 10.5 ±7.6% (plasmablasts)).

### TLR7/IL-7- increased release of IgG, IgM, and kappa free-light chains from B cells is dependent on monocytes/macrophages

**Figure 4:**
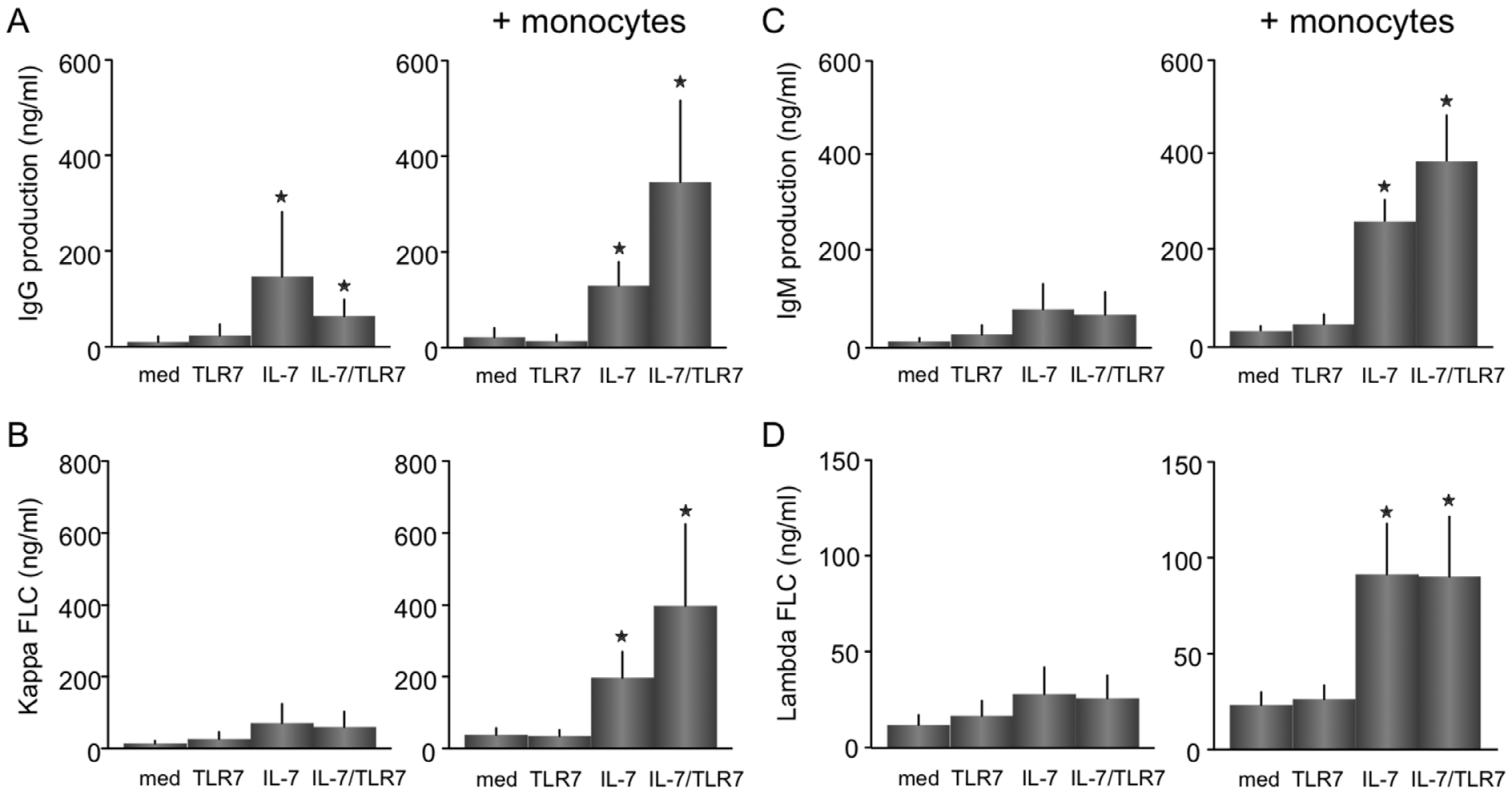
IL-7 and TLR7 increased release of IgG, IgM, and kappa free-light chains from B cells is dependent on the presence of monocytes/macrophages. The production of immunoglobulins and free-light chains was measured after a 12-day culture, cultured with or without monocytes/macrophages (n = 5). IL-7 significantly increases IgG (A), IgM (B), kappa (C) and lambda FLC (D) in the presence of monocytes/macrophages. Combined stimulation with IL-7/TLR7 induced a significant enhanced increase in the secretion of IgG (A), IgM (C), and kappa FLCs (B), only in the presence of monocytes/macrophages *p<0.05, indicates a statistical significance compared to medium values.

To further evaluate the additive effect of TLR7 and IL-7 on B cells, CD4 T cells and B cells were cultured for 12 days in the presence and absence of monocytes/macrophages after which the secretion of IgG, IgM (fig. 4A, C) and kappa and lambda FLCs (fig. 4B, D) was measured. In the absence of monocytes/macrophages, after 12 days of culture a significant increase is seen for for IgG (mean ±SEM; 11.1 ±11.1 *vs.* 147.3 ±134.8 ng/ml, p<0.05), expression upon IL-7 stimulation. No increased release of immunoglobulins was measured upon TLR7 stimulation and no additive or synergistic effect was observed upon IL-7/TLR7 stimulation. Addition of monocytes/macrophages increased IL-7-induced IgG (22.2 ±19.2 *vs*. 129.7 ±50.4 ng/ml), IgM (38.2 ±4.0 *vs*. 251.4 ±53.6 ng/ml), kappa FLCs (14.6 ±7.1 *vs.* 71.2 ±53.9 ng/ml) and lambda FLCs (23.4 ±6.7 *vs.* 91.6 ±26.1 ng/ml) (all p<0.05). Again, this was not observed upon TLR7 stimulation. IL-7 and TLR7 together enhanced the increase in IgG (346.1 ±170.6 ng/ml), IgM (202.3 ±109.9 ng/ml), and kappa FLC secretion (397.4 ±227.7 ng/ml), after 12 days of culture (all p<0.05) (fig. 4A and B).

### The combination of TLR7 and IL-7 additively increase IL-17A and synergistically increase IFNγ, IL-10 and IL-22 secretion

**Figure 5:**
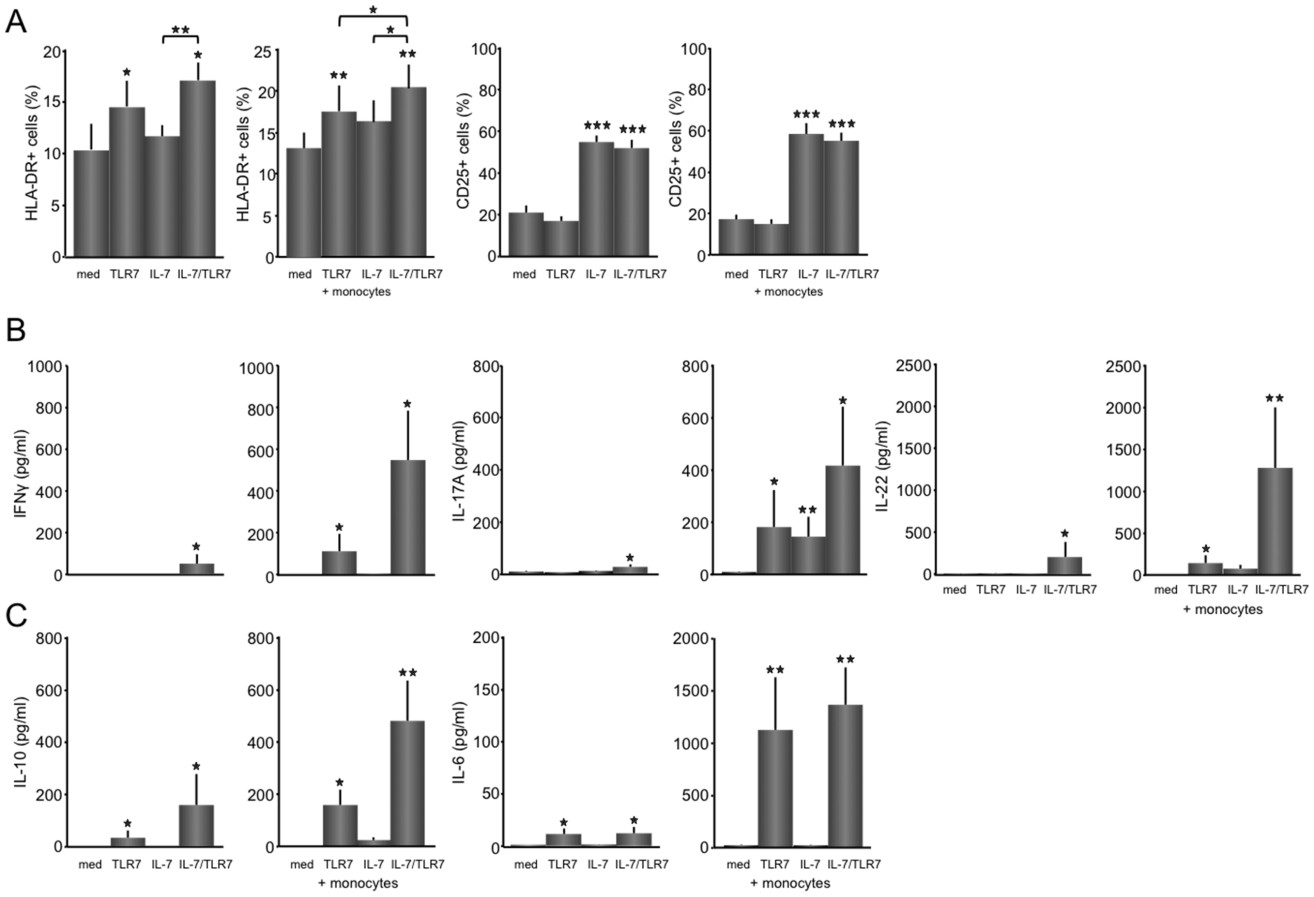
The combination of TLR7 and IL-7 additively increase IL-17A and synergistically increase IFNγ, IL-10 and IL-22 secretion. HLA-DR and CD25^+^ expression on CD4 T cells in unstimulated, TLR7, IL-7, and IL-7/TLR7 stimulated co-cultures are shown (n = 8) (A). IL-7 significant increases percentages of CD25^+^ CD4 T cells. HLA-DR^+^ CD4 T cells are increased upon TLR7 stimulation. IL-7 and TLR7A additively increase HLA-DR^+^CD4 T cells in the presence of monocytes/macrophages. No additive effect is seen for the increase in CD25^+^ CD4 T cells. IL-7 and TLR7 synergistically up-regulate Th1 and Th17-related cytokines, IFNγ, and IL-22, only in the presence of monocytes/macrophages (B). The B cell activating cytokine IL-10 is up-regulated upon TLR7 stimulation, and synergistically upon IL-7/TLR7, both in the presence and absence of monocytes, while IL-6 is potently up-regulated by TLR7 stimulation (n = 5) (C). *p<0.05, **p<0.01, and ***p<0.001 indicate statistical significance compared to medium values.

Next it was assessed whether stimulation of immunoglobulin was associated with increased expression of activation markers on CD4 T cells (fig. 5A) or cytokine production (fig. 5B, C). The percentages of activated HLA-DR-expressing T cells were increased in the presence of TLR7 and IL-7/TLR7 (mean ±SEM; medium, TLR7, IL-7 *vs.* IL-7/TLR7; 10.0 ±2.6%, 11.4 ±1.2%, 14.4 ±2.6% *vs*. 17.1 ±1.6%, p<0.05). Percentages of CD25^+^ CD4 T cells were only up regulated upon IL-7 stimulation, and were not further increased by a combination of IL-7 and TLR7 or the addition of monocytes (fig. 5A). Upon addition of monocytes/macrophages to the T/B cell cultures, the overall HLA-DR expression increased slightly and a significant (both p<0.05) additive effect of the combination *vs*. TLR7 and IL-7 monocultures was now seen (IL-7, TLR7 *vs.* IL-7/TLR7; 16.3 ±2.8%, 17.7 ±2.9% *vs*. 20.4 ±3.1%, fig. 5A).

To see whether the additive activation of B cells is associated by specific T helper cell activity major T cell-related cytokines; IFNγ, IL-2 (Th1), IL-17A, IL-21 and IL-22 (Th17), and IL-4 (Th2), were measured in supernatants of the co-cultures. In T/B co-cultures IL-4, IL-2 and IL-21 were not detected by Luminex assay, however a slight, but statistically significant increase was seen for IFNγ, IL-17A and IL-22 upon IL-7/TLR7 stimulation. In the presence of monocytes/macrophages a markedly elevated increase was seen upon TLR7 stimulation for IFNγ, IL-17A and IL-22 (med *vs*. TLR7A; IFNγ, 0.1 ±0 *vs*. 112 ±82 pg/ml; IL-17A, 9.8 ±2.7 *vs*. 182 ±142 pg/ml; IL-22, 5.5 ±3.1 *vs*. 146 ±92 pg/ml, all p<0.05) and IL-7 stimulation for IL-17A and IL-22 (med *vs.* IL-7; IL-17A, 9.8 ±2.7 *vs.* 145 ±77 pg/ml; IL-22, 5.5 ±3.1 *vs*. 78.7 ±45 pg/ml, both p<0.05). Furthermore, an additive TLR7/IL-7-induced increase was observed for the release of IL-17A (417 ±226 pg/ml, p<0.05), while IFNγ (548 ±236 pg/ml, p<0.05) and IL-22 (1284 ±721 pg/ml, p<0.01), were synergistically increased (fig. 5B).

Cytokines that are released both by B cells and monocytes/macrophages and have the potential to stimulate B cells were measured as well. IL-10 also showed a significant increase upon TLR7 stimulation in CD4 T cell/B cell co-culture without (med *vs*. TLR7A; 0.1 ±0 *vs*. 31 ±27 pg/ml, p<0.05) and with monocytes/macrophages (med *vs*. TLR7A; 1.7 ±0.7 *vs*. 159 ±59 pg/ml, p<0.05), which was synergistically increased upon the addition of IL-7, both in the absence (155 ±118 pg/ml, p<0.05) and presence (482 ±155 pg/ml, p<0.01) of monocytes/macrophages. IL-6 was potently increased by TLR7 stimulation (102 ±49 pg/ml, p<0.01) independently of IL-7 (110 ±55 pg/ml, ns), and showed a 10-fold increase (1126 ±505 pg/ml, p<0.01) when monocytes/macrophages were present (fig. 5C).

## Discussion

Here we report the synergistic effect of IL-7 and TLR7 on the proliferation and activation of both CD19 B and CD4 T cells. Furthermore, it is demonstrated that synergistic increases in immunoglobulin production are associated with synergistic induction of Th1- and Th17-related cytokines and are critically dependent on monocytes/macrophages.

Others and we have demonstrated increased numbers of Ki67-expressing proliferating lymphocytes at sites of inflammation [24], [45], [46]. In salivary glands of patients with pSS we have shown that Ki67 expression is associated with IL-7 expression [24]. In this study it is demonstrated that in co-cultures of T and B cells, IL-7 causes a profound increase in Ki67-expressing proliferating CD4 T cells, and a modest, but significant B cell proliferation. Additionally, the present data show that TLR7 stimulates B cell proliferation at a higher level, but most important IL-7 and TLR7 synergistically increases (Ki67^+^) proliferating B cells and T cells. Previously, increased expression of TLR7 in patients with pSS has also been found [47]. This suggests that both stimuli at inflammatory sites of patients with pSS, but also in other diseases, can strongly contribute to T cell and B cell proliferation.

To our knowledge the present data are also the first to demonstrate that in the presence of monocytes/macrophages IL-7-driven T cell activation and TLR7-driven B cell activation synergistically induce immunoglobulin secretion, in the absence of B cell receptor cross-linking. Cooperation of TLRs with other inflammatory mediators in the activation of B cells has been reported before, but this has mostly been in combination with BCR triggering [39], [48], [49], [50]. Overall, stimulation of the BCR together with TLRs significantly enhances B cell effector functions. The co-engagement of TLR7 and BCR has been shown to promote autoreactive B cell activity [51], [52] indicating that this could contribute to disease symptoms in autoimmune diseases. Others have shown that TLR7 can also cooperate with CD40 to enhance IL-6 secretion, independently of BCR signals [53]. However, in this latter study it has not been shown that this leads to immunoglobulin secretion. The culture system used in this study also demonstrates that in an antigen-independent environment locally overexpressed IL-7 and TLR7 [8], [16], [47], as has been shown in several autoimmune diseases, are sufficient to trigger immunoglobulin production, which makes this a relevant observation.

In association with synergistic B cell activation IL-7 and TLR7 synergistically induce Th1 and Th17 activity, which is also critically dependent on monocytes/macrophages. Surprisingly, although TLR7 hardly induces proliferation of CD4 T cells, even in the presence of monocytes/macrophages, TLR7 stimulation by itself already induced IFNγ, IL-17A and IL-22 secretion. Our data corroborate recent findings demonstrating that supernatants from TLR7-stimulated PBMC preferentially induce polarization of naive CD4 T cells towards the Th17 lineage and the release of Th17-related cytokines [54]. In addition, previously, we have shown that IL-7 strongly induces Th1, Th17 and to a lesser extent Th2 activity in co-cultures of CD4 T cells and monocytes/macrophages [6], [16], [55], [56]. In the present study, T cell cytokine secretion upon IL-7 was hardly detectable, because IL-7 secretion was not measured upon mitogenic restimulation with ionomycin/PMA or anti-CD3/CD28, as was done in our previous study and in the majority of other studies. Interestingly in this respect, without T cell restimulation, TLR7 activation together with IL-7 robustly increased expression of IFNγ, IL-17 and IL-22. In contrast to the increased induction of Th1− and Th17/Th22-related cytokines, Th2-related cytokines (IL-4) were undetectable. Although in the present study the relative contribution of these T cell cytokines to B cell activation, in particular immunoglobulin secretion, was not demonstrated, previous studies have demonstrated the capacity of Th1, Th17/Th22 cells and their cytokines to cause B cell activation including immunoglobulin secretion.

As indicated our data clearly show that the release of immunoglobulins by B cells and the production of T cell cytokines is critically dependent on CD14 monocytes/macrophages. In particular in the presence of combined IL-7 and TLR7 stimulation, the synergistic secretion of IgG and T cell cytokines was facilitated by monocytes/macrophages. Even in small numbers, like at the ratio we used in our cultures (T/B/mono; 1∶1∶0.1), monocytes/macrophages can make a distinct difference. Since monocytes themselves are hardly activated by TLR7 stimulation and they do not express the TLR7 (data not shown), effects of TLR7-activated B cells and IL-7-activated CD4 T cells could potently activate monocytes indirectly, and they in their turn can cause additional activation of B cells and T cells. Although our data do not reveal the monocyte/macrophage-driven mechanisms that are mediating the synergy of IL-7 and TLR7, they do suggest that myeloid cells, present at the site of inflammation contribute to this type of activation. In this respect, increased numbers of CD14-expressing monocytes, CD68 or CD163-expressing macrophages, CD1c or CD11c-expressing myeloid dendritic cells have been documented in different autoimmune diseases [16], [40], [45], [57], [58], [59]. The potency of these different subsets, as well as that of other potentiating cells such as plasmacytoid dendritic cells, which vastly express TLR7, remains to be demonstrated.

Elevated levels of IL-7 have been found in autoimmune diseases, like pSS [16], RA [5], JIA [60], psoriatic arthritis [8] and MS [14]. Similarly, TLRs have been linked to the pathogenesis of autoimmune disorders and increased expression of TLRs at the site of inflammation has been documented [26], [27], [28]. The results presented in this study show that combined stimulation of B cells with TLR7 agonist and IL-7-induced T cell activation synergistically enhance proliferation of B cells and CD4 T cells. This is associated with synergistically induced secretion of T helper cytokines and immunoglobulins, which are critically dependent on monocytes/macrophages. These data provide more insight into the activation and interplay of B cells and CD4 T cells and help to understand the mechanisms that play a detrimental role in autoimmune disease.
